# Knowledge, attitude, and practice of breastfeeding among mothers experiencing maternal-infant separation: a cross-sectional study

**DOI:** 10.3389/fpubh.2026.1776575

**Published:** 2026-06-16

**Authors:** Yaru Xu, Ruiqi Yan, Xi Wang, Xianmei Cui, Zhiqing Yao

**Affiliations:** 1 Gynecologic, Shanxi Bethune Hospital, Shanxi Academy of Medical Sciences, Third Hospital of Shanxi Medical University, Tongji Shanxi Hospital, Taiyuan, China; 2 Gynecologic, Xiangfen County People's Hospital, Linfen, China

**Keywords:** attitude, breastfeeding, knowledge, maternal-infant separation, practice

## Abstract

**Objective:**

Maternal-infant separation hinders breastfeeding and may reduce breastfeeding success. This study investigated breastfeeding-related knowledge, attitudes, and practices (KAP) among mothers who experienced separation.

**Methods:**

A cross-sectional study using a self-administered KAP questionnaire was conducted in Shanxi Bethune Hospital from November to December 2024.

**Results:**

A total of 513 mothers were included (effective response rate of 96.4%); 52.63% used mixed feeding and 68.76% were within 42 days postpartum. Median (interquartile ranges) scores for knowledge, attitude, and practice were 23 (22–25), 35 (32–37), and 44 (40–47), respectively. Most mothers (68.97%) reported that separation affected breastfeeding, and work environment (40.35%) and family support (38.40%) were major influencing factors. Spearman correlation analysis further revealed that knowledge was negatively correlated with attitude (r = −0.521, *P* < 0.001) and practice (r = −0.509, *P* < 0.001), whereas attitude was positively correlated with practice (r = 0.470, *P* < 0.001). In multivariable logistic regression analyses, vaginal delivery (OR = 0.624, 95% CI: 0.406–0.960) and completed maternal-infant separation (OR = 0.528, 95% CI: 0.340–0.821) were independently associated with knowledge level. Compared with those currently experiencing separation, participants who had completed separation demonstrated significantly more positive attitudes (OR = 1.815, 95% CI: 1.155–2.854) and practices (OR = 1.915, 95% CI: 1.219–3.007, *P* = 0.005).

**Conclusions:**

Mothers experiencing maternal-infant separation demonstrated limited breastfeeding knowledge but maintained positive attitudes and proactive practices. Although causal inference cannot be established in this cross-sectional study, the unexpected negative correlations between knowledge and both attitude and practice suggest that higher knowledge may reflect reactive, problem-driven learning under separation-related stress rather than a linear progression from knowledge to behavior. Targeted breastfeeding support that reinforces existing motivation may improve outcomes. Longitudinal studies are needed to clarify these relationships.

## Introduction

Maternal-infant separation has emerged as a critical barrier to successful breastfeeding, as it disrupts early mother–infant contact that is essential for establishing breastfeeding and bonding. When separation occurs, infants are deprived of the immunological protection and health benefits of human milk, placing them at greater risk of malnutrition and adverse outcomes, including infections and hospitalization (1, 2). Separation also reduces breastfeeding rates and interrupts the hormonal stimulation necessary for emotional bonding between mother and infant (3–5). As a result, minimizing maternal-infant separation has become a key strategy in global breastfeeding support programs to safeguard maternal and child health (6). However, unavoidable conditions such as preterm birth and Neonatal Intensive Care Unit (NICU) admission still require temporary separation. In China, routine NICU policies commonly separate preterm infants from their mothers, creating structural barriers to breastfeeding and contributing to low breastfeeding rates among this population (7).

The Knowledge, Attitude, and Practice (KAP) model asserts that individual behaviors are influenced by one's knowledge and attitudes. In public health, understanding these elements through KAP surveys is critical for elucidating health-related behaviors (8–10). In the context of breastfeeding, mothers' knowledge, attitudes, and practical behaviors jointly determine breastfeeding initiation, duration, and success. However, previous research highlights that, despite awareness of the importance of breastfeeding, 22% of mothers primarily rely on informal sources, such as websites, which may not align with official guidelines. Moreover, there is a significant demand among these mothers for more guidance from healthcare professionals and support from family members to maintain and effectively manage breastfeeding (11). Evidence further shows that mothers who experience maternal-infant separation, particularly those with preterm infants in NICUs, face greater challenges in acquiring accurate breastfeeding knowledge and skills outside the hospital environment, often leading to uncertainty in milk expression, storage, and continued breastfeeding (11, 12).

Investigating the KAP among mothers experiencing maternal-infant separation is essential for identifying the key barriers and facilitators to breastfeeding in this context. Although breastfeeding is particularly critical for improving health outcomes in preterm infants, existing studies primarily focus on breastfeeding outcomes, such as breastfeeding rates, while comprehensive analyses of knowledge, attitudes, and practices, as well as their influencing factors, among separated mothers remain scarce. Therefore, this study aims to examine breastfeeding-related KAP among mothers undergoing maternal-infant separation to inform tailored interventions that enhance breastfeeding support and outcomes. It was hypothesized that mothers experiencing maternal-infant separation would demonstrate a mismatch between breastfeeding knowledge and behavioral practices under separation-related stress, and that separation stage might influence KAP patterns. Beyond traditional breastfeeding outcome studies that primarily focus on rates or duration, the present study emphasizes the behavioral patterns underlying breastfeeding decisions in separation-related contexts. By examining how knowledge, attitudes, and practices interact under stress conditions, this approach seeks to provide a more nuanced understanding of breastfeeding behavior and to inform interventions that address not only outcomes but also the mechanisms driving those outcomes.

## Methods

### Study design and subjects

This cross-sectional study was conducted from November 2024 to December 2024 in Shanxi Bethune Hospital, targeting breastfeeding mothers. The study was approved by the Medical Ethics Committee of Shanxi Bethune Hospital (Approval No. YXLL-2024-205), and informed consent was obtained electronically before participants completed the questionnaire.

Breastfeeding mothers who experienced maternal-infant separation for more than 24 h within the first 6 months postpartum and who provided informed consent to voluntarily participate in the survey were included in the study, whereas those whose separation lasted less than 24 h were excluded.

### Procedures

The questionnaire was developed with reference to relevant guidelines and existing literature, and its content validity was strengthened through a multi-step expert review and pilot testing process. Initially drafted by the research team, the questionnaire underwent evaluation by three obstetric specialists (the head of the obstetrics department, the chief nursing officer of obstetrics and gynecology, and the head nurse of the obstetric ward) and three additional obstetrics colleagues, whose feedback informed revisions to form the preliminary version. Two rounds of small-scale pilot testing were subsequently conducted, and further adjustments were made based on the pre-test results. The final pre-test demonstrated excellent internal consistency, with a Cronbach's α of 0.982 for the total scale (knowledge: 0.955; attitude: 0.969; practice: 0.946).

The finalized questionnaire consisted of four sections: (1) demographic characteristics (age, education level, mode of delivery, postpartum period, and maternal-infant separation status); (2) knowledge; (3) attitude; and (4) practice. The knowledge section contained 15 items on the benefits of breastfeeding for both the mother and infant, as well as factors influencing milk secretion. All items were positively worded and rated on a three-point scale (“very clear” = 3, “heard of it” = 2, “never heard of it” = 1), yielding a total score range of 15–45. The attitude section included 10 items. Two items (A5 and A7) were open-ended, exploring the negative impact of maternal-infant separation on breastfeeding and environmental influences on breastfeeding decisions, respectively. The remaining items were rated on a five-point Likert scale from “strongly positive” (5) to “strongly negative” (1), with a total score range of 8–40. The practice section consisted of 13 items. One item (P9) was open-ended to explore reasons for delayed initiation of breastfeeding and was not scored. Items P2 and P3 assessed breastfeeding decisions before and after maternal-infant separation (breastfeeding = 3; formula feeding = 1; mixed feeding = 2), while the remaining items adopted a five-point Likert scale from “always” (5) to “never” (1), with a total score range of 13–61.

The questionnaire was developed using the WeChat mini-program provided by Questionnaire Star and distributed through QR codes shared in patient WeChat groups and hospital wards. To encourage honest responses and protect participant privacy, all data were collected anonymously. To ensure data integrity, each IP address was limited to a single submission, and all questions were required to be answered. The research team meticulously reviewed all responses to verify completeness, internal consistency, and logical coherence.

### Sample size calculation

We determined the minimum required sample size (n) using the standard formula for cross-sectional studies, assuming the maximum sample size where the proportion (p) is 0.5:


n=Z(1−α)/2p2(1−p)δ2 (1)

The Type I error (α) was set to 0.05, corresponding to a Z-score of 1.96 (for a 95% confidence level). The standard error (δ) was set at 0.05. Substituting these values yields a required sample size of 384. Assuming an effective questionnaire recovery rate of 90%, the final target is to collect at least 430 completed questionnaires.

### Statistical analysis

Descriptive statistics summarized participant characteristics and KAP scores, using medians with interquartile ranges (IQR) for non-normally distributed continuous variables and n (%) for categorical data. Group differences were assessed via Mann–Whitney U or Kruskal–Wallis tests, while Spearman correlation analyzed associations between KAP dimensions. Knowledge, attitude, and practice were dichotomized as “good,” “positive,” or “proactive” based on a 60% total score cutoff for logistic regression. Multicollinearity was screened using Variance Inflation Factor (VIF); notably, feeding method was excluded from the multivariate attitude model due to significant collinearity (VIF > 10). To ensure robustness, sensitivity analyses using multivariable linear regression were performed on continuous scores. Statistical significance was defined as two-tailed *P* < 0.05$.

## Results

### Participant demographics

A total of 532 questionnaires were collected, of which 19 were excluded for being completed in less than 90 s, resulting in 513 valid responses and an effective response rate of 96.4%. Among the 513 participants, 226 (44.05%) were aged 30–35 years, 252 (49.12%) had a Bachelor's degree, 340 (66.28%) had 1 child, 347 (67.64%) had vaginal delivery, 374 (72.90%) have experienced maternal-infant separation and have completed. The median (IQR) for knowledge, attitude, and practice scores were 23 (possible range: 15–45), 35 (possible range: 10–50), and 44 (possible range: 13–65), respectively (Table 1).

**Table 1:** Basic information of participants and KAP score.

Characteristics	*N* (%)	Knowledge, median (IQR)	*P*	Attitude, median (IQR)	*P*	Practice, median (IQR)	*P*
*N* = 513							
**Total score**		23 (22–25)		35 (32–37)		44 (40–47)	
Age							
< 25 years old	83 (16.18%)	23 (22–25)	0.129	35 (33–37)	0.642	45 (42–47)	0.005
25 to < 30 years old	226 (44.05%)	23 (22–35)		35 (24–38)		43 (28–46)	
30 to < 35years old	125 (24.37%)	23 (21–14)		36 (33–37)		45 (42–47)	
≥35 years old	79 (15.40)	23 (21–28)		36 (32–38)		44 (39–47)	
Education level							
High school or below	39 (7.60%)	24 (22–28)	0.378	35 (32–37)	0.824	43 (40–46)	0.378
Associate degree	139 (27.10%)	23 (21–28)		36 (30–37)		44 (39–47)	
Bachelor's degree	252 (49.12%)	23 (21–25)		35 (32–37)		44 (38.25–47)	
Master's degree	83 (16.18%)	23 (22–25)		35 (33–37)		44 (42–47)	
Your current employment status							
Housewife	57 (11.11%)	23 (22–27)	0.736	35 (31–37)	0.160	44 (39–47)	0.947
Employed	322 (62.77%)	23 (21–25)		36 (33–37)		44 (40–47)	
Self-employed	114 (22.22%)	23 (22–34)		35 (25–37)		44.5 (32.75–47)	
Other	20 (3.90%)	23.5 (21.25–28)		35.5 (30–37.75)		44 (37–45.75)	
Household's disposable income (annual)							
80,000 yuan or above	243 (47.37%)	23 (22–25)	0.897	36 (33–37)	0.566	45 (41–47)	0.031
40,000 to < 80,000 yuan	163 (31.77%)	23 (22–27)		35 (28–37)		43 (34–46)	
< 40,000 yuan	107 (20.86%)	23 (22–28)		35 (30–37)		44 (38–46)	
How many children do you have?							
1	340 (66.28%)	23 (22–25)	0.860	36 (32–37)	0.383	44 (40–47)	0.678
2 or more	173 (33.72%)	23 (22–26.5)		35 (32–37)		44 (39–47)	
Your mode of delivery							
Vaginal delivery	347 (67.64%)	23 (21–25)	0.063	36 (33–37)	0.492	45 (41–47)	0.009
Cesarean section	166 (32.36%)	23 (22–35)		35 (24.75–37.25)		43 (27.75–46)	
Your current feeding method							
Exclusive breastfeeding	229 (44.64%)	23 (21–25)	< 0.001	36 (34–38)	< 0.001	44 (41–47)	< 0.001
Mixed feeding	270 (52.63%)	23 (22–33)		35 (26.5–37)		44 (31.75–47)	
Formula feeding	14 (2.73%)	41 (35.5–45)		19 (15–22.5)		23.5 (21.75–27.75)	
Current postpartum period							
≤ 3 days	67 (13.06%)	23 (21–25)	0.640	36 (33–37)	0.065	45 (41–47)	0.082
>3 to 7 days	178 (34.70%)	23 (21.75–25)		35 (32–37)		44 (41–47)	
>7 to 42 days	108 (21.05%)	23 (22–25)		35 (33–37)		45 (41.25–47)	
>42 days to 6 months	89 (17.35%)	24 (22–36)		35 (24–38)		43 (29.5–46)	
Greater than 6 months	71 (13.84%)	23 (22–26)		37 (32–39)		43 (37–46)	
What stage are you currently in?							
Already experienced maternal-infant separation; currently, the separation has ended	374 (72.90%)	23 (22–25)	0.061	35 (33–37)	0.014	45 (41–47)	< 0.001
Currently experiencing maternal-infant separation; the separation has not yet ended	139 (27.10%)	24 (21–35)		(21–37)		43 (27–46)	

### Knowledge, attitude, and practice scores

The distribution of knowledge dimensions shown that the three questions with the highest number of participants choosing the “Never heard of it” option were “Can a mother continue breastfeeding her baby if she has the flu or a severe cold?” (K7) with 12.28%, “Breast milk contains sufficient energy and nutrients, which are more easily digested and absorbed.” (K2) with 11.89%, and “Can exclusive breastfeeding positively impact the mother's own emotions and feelings and elicit positive emotional responses in others?” (K12) with 11.89% (Supplementary Table S1).

Responses to the attitude dimension showed that 35.28% strongly agreed, 33.72% agreed, with the item that “Has maternal-infant separation negatively impacted your ability to breastfeed?” (A5). When it comes to environmental factors that affect their decision breastfeeding decision (A7), 40.35% believe that it is the work environment, 38.40% believe that it is family members, 9.94% believe that it is surrounding people, and 11.31% believe that it is public places (Supplementary Table S2).

When it comes to related attitudes, the feeding method that 44.05% of the participants communicated and confirmed with their families in advance before delivery was breastfeeding (P2), and after the end of maternal-infant separation, 40.94% of the participants opted for or wished for breastfeeding (P3). However, when it comes to the reasons for not allowing their babies to breastfeed in time, 77.58% were due to the fact that their physical condition did not allow them to breastfeed (P9) (Supplementary Table S3).

### Correlation analysis

Further correlation analysis revealed negative correlations between knowledge scores and attitude scores (r = −0.521, *P* < 0.001), as well as between knowledge scores and practice scores (r = −0.509, *P* < 0.001). Additionally, attitude scores were positively correlated with practice scores (r = 0.470, *P* < 0.001) (Table 2).

**Table 2:** Correlation analysis of KAP scores.

Variables	Knowledge	Attitude	Practice
Knowledge	1		
Attitude	−0.521 (*P* < 0.001)	1	
Practice	−0.509 (*P* < 0.001)	0.470 (*P* < 0.001)	1

### Univariate and multivariate analysis

Multivariate logistic regression analysis identified several factors significantly associated with knowledge scores. Vaginal delivery (OR = 0.624, 95% CI: 0.406–0.960, *P* = 0.032) and completed maternal-infant separation (OR = 0.528, 95% CI: 0.340–0.821, *P* = 0.005) remained independently associated with knowledge level after adjustment (Table 3). In the attitude dimension, completed maternal-infant separation was independently associated with more positive attitudes (OR = 1.815, 95% CI: 1.155–2.854, *P* = 0.010) (Table 4). For the practice dimension, already experienced maternal-infant separation (OR = 1.915, 95% CI: 1.219–3.007, *P* = 0.005) independently associated with proactive breastfeeding practice in the multivariable model (Table 5). Sensitivity analyses using continuous KAP scores in multivariable linear regression models yielded results generally consistent with those of the logistic regression analyses (Supplementary Tables S4–6), supporting the robustness of the findings.

**Table 3:** Univariable and multivariable logistic regression for knowledge dimension.

Knowledge	Univariate logistic regression		Multivariate logistic regression	
	OR (95% CI)	*P*	OR (95% CI)	*P*
Age				
< 25 years old	0.513 (0.236–1.113)	0.091		
25 to < 30 years old	1.139 (0.641–2.026)	0.657		
30 to < 35years old	0.465 (0.229–0.941)	0.033		
≥35 years old	ref			
Education level				
High school or below	1.700 (0.677–4.265)	0.259		
Associate degree	1.854 (0.935–3.678)	0.077		
Bachelor's degree	1.408 (0.738–2.689)	0.300		
Master's degree or above	ref			
Your current employment status				
Housewife	0.760 (0.245–2.353)	0.634		
Employed	0.602 (0.233–1.625)	0.316		
Self-employed	0.911 (0.322–2.576)	0.860		
Other	ref			
Household's disposable income (annual)				
80,000 yuan or above	0.677 (0.390–1.156)	0.153		
40,000 to < 80,000 yuan	1.011 (0.581–1.760)	0.969		
< 40,000 yuan	ref			
How many children do you have?				
1	0.856 (0.557–1.315)	0.477		
2 or more	ref			
Your mode of delivery				
Vaginal delivery	0.593 (0.387–0.907)	0.016	0.624 (0.406–0.960)	0.032
Cesarean section	ref		ref	
Your current feeding method				
Exclusive breastfeeding	0.029 (0.006–0.136)	< 0.001		
Mixed feeding	0.061 (0.013–0.277)	< 0.001		
Formula feeding	ref			
Current postpartum period				
≤ 3 days	ref			
>3 to 7 days	1.028 (0.516–2.048)	0.938		
>7 to 42 days	1.024 (0.485–2.164)	0.950		
>42 days to 6 months	1.649 (0.785–3.464)	0.187		
Greater than 6 months	1.101 (0.490–2.477)	0.815		
What stage are you currently in?				
Already experienced maternal-infant separation; currently, the separation has ended	0.507 (0.327–0.785)	0.002	0.528 (0.340–0.821)	0.005
Currently experiencing maternal-infant separation; the separation has not yet ended	ref		ref	

**Table 4:** Univariable and multivariable logistic regression for attitude dimension.

Attitude	Univariate logistic regression		Multivariate logistic regression	
	OR (95% CI)	*P*	OR (95% CI)	*P*
Age				
< 25 years old	1.589 (0.720–3.507)	0.252		
25 to < 30 years old	0.798 (0.437–1.458)	0.463		
30 to < 35years old	1.875 (0.900–3.903)	0.093		
≥35 years old	ref			
Education level				
High school or below	0.619 (0.239–1.603)	0.323		
Associate degree	0.621 (0.305–1.265)	0.189		
Bachelor's degree	0.665 (0.343–1.291)	0.228		
Master's degree or above	ref			
Your current employment status				
Housewife	0.597 (0.151–2.361)	0.462		
Employed	0.711 (0.202–2.502)	0.596		
Self-employed	0.517 (0.141–1.894)	0.319		
Other	ref			
Household's disposable income (annual)				
80,000 yuan or above	1.272 (0.728–2.222)	0.397		
40,000 to < 80,000 yuan	0.889 (0.499–1.584)	0.690		
< 40,000 yuan	ref			
How many children do you have?				
1	1.013 (0.648–1.583)	0.956		
2 or more	ref			
Your mode of delivery				
Vaginal delivery	1.565 (1.010–2.424)	0.045	1.487 (0.956–2.315)	0.079
Cesarean section	ref		ref	
Your current feeding method				
Exclusive breastfeeding	24.322 (6.413–92.246)	< 0.001		
Mixed feeding	10.892 (2.951–40.201)	< 0.001		
Formula feeding	ref			
Current postpartum period				
≤ 3 days	ref			
>3 to 7 days	1.007 (0.504–2.010)	0.985		
>7 to 42 days	1.162 (0.542–2.493)	0.699		
>42 days to 6 months	0.715 (0.337–1.518)	0.383		
Greater than 6 months	1.075 (0.469–2.466)	0.864		
What stage are you currently in?				
Already experienced maternal-infant separation; currently, the separation has ended	1.883 (1.201–2.951)	0.006	1.815(1.155–2.854)	0.010
Currently experiencing maternal-infant separation; the separation has not yet ended	ref		ref	

**Table 5:** Univariable and multivariable logistic regression for practice dimension.

Practice	Univariate logistic regression		Multivariate logistic regression	
	OR (95% CI)	*P*	OR (95% CI)	*P*
Age				
< 25 years old	1.589 (0.720–3.507)	0.252		
25 to < 30 years old	0.781 (0.428–1.424)	0.419		
30 to < 35years old	2.010 (0.956–4.225)	0.065		
≥35 years old	ref			
Education level				
High school or below	0.619 (0.239–1.603)	0.323		
Associate degree	0.621 (0.305–1.265)	0.189		
Bachelor's degree	0.665 (0.343–1.291)	0.665		
Master's degree or above	ref			
Your current employment status				
Housewife	0.597 (0.151–2.361)	0.462		
Employed	0.711 (0.202–2.502)	0.596		
Self–employed	0.517 (0.141–1.894)	0.319		
Other	ref			
Household's disposable income (annual)				
80,000 yuan or above	1.272 (0.728–2.222)	0.397		
40,000 to < 80,000 yuan	0.889 (0.499–1.584)	0.690		
< 40,000 yuan	ref			
How many children do you have?				
1	1.272 (0.728–2.222)	0.397		
2 or more	0.889 (0.499–1.584)	0.690		
Your mode of delivery				
Vaginal delivery	1.565 (1.010–2.424)	0.045	1.487(0.951–2.306)	0.079
Cesarean section	ref		ref	
Your current feeding method				
Exclusive breastfeeding	24.322 (6.413–92.246)	< 0.001		
Mixed feeding	10.892 (2.951–40.201)	< 0.001		
Formula feeding	ref			
Current postpartum period				
≤ 3 days	ref			
>3 to 7 days	0.917 (0.453–1.858)	0.811		
>7 to 42 days	1.059 (0.487–2.302)	0.884		
>42 days to 6 months	0.616 (0.288–1.320)	0.213		
Greater than 6 months	0.980 (0.422–2.274)	0.963		
What stage are you currently in?				
Already experienced maternal-infant separation; currently, the separation has ended	1.984 (1.267–3.106)	0.003	1.915(1.219–3.007)	0.005
Currently experiencing maternal-infant separation; the separation has not yet ended	ref		ref	

## Discussion

This study found that although mothers who experienced maternal-infant separation demonstrated relatively low levels of breastfeeding knowledge, their attitudes and practices remained generally positive. Most mothers expressed support for breastfeeding and made active efforts to initiate or maintain breastfeeding during separation or after reunification, indicating a strong intrinsic motivation for breastfeeding. These findings suggest that in the context of maternal-infant separation, mothers' breastfeeding attitudes and behaviors are not associated with their level of formal breastfeeding knowledge but are also closely linked to emotional factors, personal experience, family support, cultural expectations, and clinical guidance.

A notable finding of this study is the significant negative correlation between knowledge and both attitude and practice, which deviates from the traditional KAP model that assumes a linear pathway from knowledge to behavior (8–10). One possible explanation is that mothers with higher knowledge scores may have faced more complex breastfeeding challenges, such as preterm birth, NICU hospitalization, or feeding difficulties, prompting them to proactively seek more information and thereby achieving higher knowledge scores (11, 12). This pattern may be conceptualized as “reactive learning,” whereby knowledge acquisition is driven by the experience of breastfeeding difficulties rather than preceding them. In separation-related contexts, mothers encountering challenges such as preterm birth, delayed lactation, or prolonged separation may engage in intensive information-seeking behaviors, resulting in higher knowledge scores. In contrast, mothers experiencing smoother postpartum recovery or shorter separation may rely more on intuition, prior informal experience, or immediate support from healthcare providers and family, thereby demonstrating positive practices despite relatively lower formal knowledge. However, the stressful circumstances associated with these conditions may hinder the development of positive attitudes or consistent breastfeeding practices (11, 12). In contrast, mothers who experienced shorter separation, smoother postpartum recovery, or stronger family support may have maintained more positive attitudes and behaviors despite possessing limited formal knowledge. These results imply that contextual stressors and structural constraints may be more strongly associated with attitudes and behaviors than knowledge alone in separation-related situations (13, 14). This hypothesis could be further tested in longitudinal studies by examining temporal sequences between the onset of breastfeeding challenges, subsequent information-seeking behaviors, and changes in knowledge, attitudes, and practices over time.

Multivariable analysis suggested that feeding-related behaviors were associated with breastfeeding practice, although effect sizes were moderate after adjustment. The observed associations likely reflect behavioral alignment between feeding intentions and reported practices rather than independent causal effects. In maternal-infant separation contexts, breastfeeding behavior may be shaped by emotional attachment, perceived maternal responsibility, and social support mechanisms (15, 16). These psychosocial influences, together with structural and environmental factors, may contribute to consistent behavioral patterns beyond knowledge acquisition alone (15–17).

This study also found that the end of maternal-infant separation was associated with more positive attitudes and improved breastfeeding practices, though knowledge levels did not show a corresponding increase. This suggests that current clinical practices may emphasize practical support and emotional encouragement more than structured breastfeeding education. Previous literature indicates that mothers separated from their infants—particularly those with NICU experiences—often lack access to systematic knowledge about milk expression, storage, and lactation maintenance outside the hospital setting (11, 12). Meanwhile, family members and workplace environments were identified in our study as key influences on breastfeeding decisions, consistent with evidence showing that environmental and social support can compensate for inadequate knowledge and help sustain breastfeeding behaviors (13, 18, 19).

From a public health perspective, the pattern of “low knowledge but positive attitudes and practices” presents both opportunities and risks. On one hand, strong breastfeeding motivation provides a favorable foundation for interventions. On the other hand, insufficient knowledge may increase the risk of poor lactation management, reduced milk supply, or early breastfeeding cessation—issues commonly observed among mothers with inconsistent breastfeeding guidance (20–27). Therefore, interventions should prioritize strengthening evidence-based breastfeeding education while preserving mothers' naturally positive attitudes and behaviors. Importantly, educational programs should move beyond knowledge transmission alone and align more closely with mothers' existing motivation and positive practices. Integrating emotional support, confidence-building strategies, and structured lactation coaching may help translate strong breastfeeding intentions into sustainable behaviors. By reinforcing maternal self-efficacy and leveraging family and workplace support systems, interventions may more effectively bridge the gap between knowledge and practice in separation-related contexts. Prior research supports the use of structured prenatal counseling (18, 19), enhanced post-discharge support for separated mothers (11, 12, 28), and community-based continuity-of-care models, all of which may improve breastfeeding sustainability in populations experiencing maternal-infant separation.

This study has several limitations. First, its cross-sectional design prevents causal inference, particularly regarding the directionality of the observed negative associations between knowledge and behavior. Second, the use of self-administered questionnaires may introduce recall and social desirability biases. However, these biases were partially mitigated through anonymous data collection, mandatory completion settings to minimize missing data, and rigorous logical consistency checks during data screening. Third, although the questionnaire underwent expert review, certain knowledge items may not fully capture the breadth and depth of breastfeeding knowledge. In addition, although the KAP framework provides a structured approach to examining health-related behaviors, it may not fully capture the complexity of breastfeeding behavior under maternal-infant separation. In high-stress contexts, behavioral responses may also be shaped by coping strategies, perceived self-efficacy, emotional regulation, and social support dynamics. Complementary theoretical perspectives, such as coping theory or self-efficacy theory, may therefore offer additional explanatory value. Furthermore, this study did not collect detailed data on the exact duration (in days) or the specific medical reasons for maternal-infant separation, precluding subgroup analyses to explore heterogeneity in KAP patterns according to separation characteristics. Future research should incorporate these detailed variables, adopt longitudinal designs, include objective breastfeeding indicators (e.g., direct breastfeeding rates, milk volume, lactation duration), and potentially integrate qualitative approaches to more comprehensively explore the multidimensional mechanisms underlying breastfeeding behavior in separation-related contexts.

## Conclusion

Mothers who experienced maternal-infant separation showed limited breastfeeding knowledge but maintained positive attitudes and proactive breastfeeding practices. Although the cross-sectional design precludes causal inference and reverse causality cannot be excluded, the observed negative associations suggest that breastfeeding behaviors in this context may not follow a simple linear progression from knowledge to practice. Higher knowledge scores may reflect reactive, problem-driven information seeking triggered by breastfeeding challenges during separation. Strengthening structured breastfeeding education, while reinforcing mothers' existing motivation, emotional resilience, and supportive environments, may help improve breastfeeding outcomes for separated mothers. Future longitudinal and intervention-based studies are planned to further validate these findings and examine how targeted support strategies can optimize breastfeeding behaviors in maternal-infant separation settings.

## Data Availability

The original contributions presented in the study are included in the article/Supplementary material, further inquiries can be directed to the corresponding author.

## References

[1] Eidelman AI. Breastfeeding and the use of human milk: an analysis of the American Academy of Pediatrics 2012 Breastfeeding Policy Statement. Breastfeed Med. (2012) 7:323–4. doi: 10.1089/bfm.2012.0067 22946888

[2] Victora CG Bahl R Barros AJ França GV Horton S Krasevec J . Breastfeeding in the 21st century: epidemiology, mechanisms, and lifelong effect. Lancet. (2016) 387:475–90. doi: 10.1016/S0140-6736(15)01024-7 26869575

[3] Conti MG Natale F Stolfi I Pedicino R Boscarino G Ajassa C . Consequences of early separation of maternal-newborn dyad in neonates born to SARS-CoV-2 positive mothers: an observational study. Int J Environ Res Public Health. (2021) 18:5899. doi: 10.3390/ijerph18115899 34072815 PMC8199070

[4] Moore ER Bergman N Anderson GC Medley N. Early skin-to-skin contact for mothers and their healthy newborn infants. Cochrane Database Syst Rev. (2016) 11:Cd003519. doi: 10.1002/14651858.CD003519.pub4 27885658 PMC6464366

[5] Tomori C Gribble K Palmquist AEL Ververs MT Gross MS. When separation is not the answer: Breastfeeding mothers and infants affected by COVID-19. Matern Child Nutr. (2020) 16:e13033. doi: 10.1111/mcn.13033 32458558 PMC7267086

[6] WHO Guidelines Approved by the Guidelines Review Committee. Guideline: Protecting, Promoting and Supporting Breastfeeding in Facilities Providing Maternity and Newborn Services. Geneva: World Health Organization (2017). Copyright © World Health Organization 2017. 29565522

[7] Hei M Gao X Gao X Nong S Zhang A Zhang Q . Is family integrated care in neonatal intensive care units feasible and good for preterm infants in China: study protocol for a cluster randomized controlled trial. Trials. (2016) 17:22. doi: 10.1186/s13063-015-1152-9 26758621 PMC4711020

[8] Aerts C Revilla M Duval L Paaijmans K Chandrabose J Cox H . Understanding the role of disease knowledge and risk perception in shaping preventive behavior for selected vector-borne diseases in Guyana. PLoS Negl Trop Dis. (2020) 14:e0008149. doi: 10.1371/journal.pntd.0008149 32251455 PMC7170267

[9] Liao L Feng H Jiao J Zhao Y Ning H. Nursing assistants' knowledge, attitudes and training needs regarding urinary incontinence in nursing homes: a mixed-methods study. BMC Geriatr. (2023) 23:39. doi: 10.1186/s12877-023-03762-z 36683023 PMC9867858

[10] Mumena WA. Maternal knowledge, attitude and practices toward free sugar and the associations with free sugar intake in children. Nutrients (2021) 13:4403. doi: 10.3390/nu13124403 34959955 PMC8706702

[11] Yang R Chen D Wang H Xu X. Experiences of mothers of NICU preterm infants in milk management out of the hospital: a qualitative study. Int Breastfeed J. (2022) 17:95. doi: 10.1186/s13006-022-00540-2 36587203 PMC9805215

[12] Yang Y Brandon D Lu H Cong X. Breastfeeding experiences and perspectives on support among Chinese mothers separated from their hospitalized preterm infants: a qualitative study. Int Breastfeed J. (2019) 14:45. doi: 10.1186/s13006-019-0242-9 31695726 PMC6824106

[13] Halili L Liu RH Weeks A Deonandan R Adamo KB. High maternal self-efficacy is associated with meeting institute of medicine gestational weight gain recommendations. PLoS ONE. (2019) 14:e0226301. doi: 10.1371/journal.pone.0226301 31826008 PMC6905531

[14] Speranzini N Goodarzi Z Casselman L Pringsheim T. Barriers and facilitators associated with the management of aggressive and disruptive behaviour in children: a qualitative study with pediatricians. J Can Acad Child Adolesc Psychiatry. (2020) 29:177–87. 32774400 PMC7391873

[15] Emmott EH Page AE Myers S. Typologies of postnatal support and breastfeeding at two months in the UK. Soc Sci Med. (2020) 246:112791. doi: 10.1016/j.socscimed.2020.112791 31927156 PMC7014584

[16] Nurokhmah S Rahmawaty S Puspitasari DI. Determinants of Optimal Breastfeeding Practices in Indonesia: findings From the 2017 Indonesia Demographic Health Survey. J Prev Med Public Health. (2022) 55:182–92. doi: 10.3961/jpmph.21.448 35391530 PMC8995937

[17] Pezley L Cares K Duffecy J Koenig MD Maki P Odoms-Young A . Efficacy of behavioral interventions to improve maternal mental health and breastfeeding outcomes: a systematic review. Int Breastfeed J. (2022) 17:67. doi: 10.1186/s13006-022-00501-9 36064573 PMC9446548

[18] Kehinde J O'Donnell C Grealish A. The effectiveness of prenatal breastfeeding education on breastfeeding uptake postpartum: a systematic review. Midwifery. (2023) 118:103579. doi: 10.1016/j.midw.2022.103579 36580847

[19] Shafaei FS Mirghafourvand M Havizari S. The effect of prenatal counseling on breastfeeding self-efficacy and frequency of breastfeeding problems in mothers with previous unsuccessful breastfeeding: a randomized controlled clinical trial. BMC Womens Health. (2020) 20:94. doi: 10.1186/s12905-020-00947-1 32370804 PMC7201717

[20] Ambaw YL Yirdaw BW Biwota MA Mekuryaw AM Taye BT. Antenatal care follow-up decreases the likelihood of cultural malpractice during childbirth and postpartum among women who gave birth in the last one-year in Gozamen district, Ethiopia: a community-based cross-sectional study. Arch Public Health. (2022) 80:53. doi: 10.1186/s13690-022-00814-5 35168678 PMC8845281

[21] Chea N Asefa A. Prelacteal feeding and associated factors among newborns in rural Sidama, south Ethiopia: a community based cross-sectional survey. Int Breastfeed J. (2018) 13:7. doi: 10.1186/s13006-018-0149-x 29467812 PMC5819158

[22] Panahi F Rashidi Fakari F Nazarpour S Lotfi R Rahimizadeh M Nasiri M . Educating fathers to improve exclusive breastfeeding practices: a randomized controlled trial. BMC Health Serv Res. (2022) 22:554. doi: 10.1186/s12913-022-07966-8 35468827 PMC9040207

[23] Abukari AS Acheampong AK. Breastfeeding practices and coping strategies adopted by lactating nurses and midwives: a qualitative study. J Pediatr Nurs. (2022) 66:e61–e6. doi: 10.1016/j.pedn.2022.05.017 35637105

[24] Horwood C Haskins L Engebretsen I Connolly C Coutsoudis A Spies L. Are we doing enough? Improved breastfeeding practices at 14 weeks but challenges of non-initiation and early cessation of breastfeeding remain: findings of two consecutive cross-sectional surveys in KwaZulu-Natal, South Africa. BMC Public Health. (2020) 20:440. doi: 10.1186/s12889-020-08567-y 32245371 PMC7118904

[25] Horwood C Surie A Haskins L Luthuli S Hinton R Chowdhury A . Attitudes and perceptions about breastfeeding among female and male informal workers in India and South Africa. BMC Public Health. (2020) 20:875. doi: 10.1186/s12889-020-09013-9 32503486 PMC7275335

[26] Linde K Lehnig F Nagl M Kersting A. The association between breastfeeding and attachment: a systematic review. Midwifery. (2020) 81:102592. doi: 10.1016/j.midw.2019.102592 31830673

[27] Walsh A Pieterse P Mishra N Chirwa E Chikalipo M Msowoya C . Improving breastfeeding support through the implementation of the baby-friendly hospital and community initiatives: a scoping review. Int Breastfeed J. (2023) 18:22. doi: 10.1186/s13006-023-00556-2 37061737 PMC10105160

[28] Fan HSL Ho MY Ko RWT Kwok JYY Chau PH Wong JYH . Feasibility and effectiveness of WhatsApp online group on breastfeeding by peer counsellors: a single-blinded, open-label pilot randomized controlled study. Int Breastfeed J. (2022) 17:91. doi: 10.1186/s13006-022-00535-z 36544208 PMC9771777

