# Supplementary material for: Knowledge, attitude, and practice of breastfeeding among mothers experiencing maternal-infant separation: a cross-sectional study
Source: Front Public Health. 2026 Jun 16;14:1776575. doi: 10.3389/fpubh.2026.1776575 (PMC13315230; doi:10.3389/fpubh.2026.1776575)
Supplement: Supplementary file 1 [file Data_Sheet_1.docx]

Supplementary table 1. Knowledge dimension of the participants.

| Knowledge | **N (%)** | | |
| --- | --- | --- | --- |
|  | **Very clear** | **Heard of it** | **Never heard of it** |
| 1. Exclusive breastfeeding is the best food for newborns. | 213 (41.52%) | 247 (48.15%) | 53 (10.33%) |
| 1. Breast milk contains sufficient energy and nutrients, which are more easily digested and absorbed. | 211 (41.13%) | 241 (46.98%) | 61 (11.89%) |
| 1. The optimal feeding method for infants is exclusive breastfeeding from birth to 6 months, continuing breastfeeding up to 2 years or beyond. | 207 (40.35%) | 247 (48.15%) | 59 (11.50%) |
| 4. Breastfeeding can enhance the emotional bond between the child and the mother, providing warmth and love to the child. | 196 (38.21%) | 259 (50.49%) | 58 (11.31%) |
| 5. Can breastfeeding improve a child’s immunity? | 206 (40.16%) | 253 (49.32%) | 54 (10.53%) |
| 6. Breastfed children are less likely to develop diabetes, heart disease, eczema, asthma, rheumatoid arthritis, and other allergic diseases, and breastfeeding can prevent obesity. | 227 (44.25%) | 226 (44.05%) | 60 (11.70%) |
| 7. Can a mother continue breastfeeding her baby if she has the flu or a severe cold? | 204 (39.77%) | 246 (47.95%) | 63 (12.28%) |
| 8. Breastfeeding can reduce the risk of breast and ovarian cancer in women. | 199 (38.79%) | 255 (49.71%) | 59 (11.50%) |
| 9. Breastfeeding can reduce postpartum bleeding and anemia, promoting faster recovery after childbirth. | 207 (40.35%) | 251 (48.93%) | 55 (10.72%) |
| 10. Breastfeeding mothers are less likely to be obese; breastfeeding helps mothers return to their normal body shape. | 193 (37.62%) | 269 (52.44%) | 51 (9.94%) |
| 11. Exclusive breastfeeding has a contraceptive effect, inhibiting ovulation and delaying the return of fertility. | 211 (41.13%) | 242 (47.17%) | 60 (11.70%) |
| 12. Can exclusive breastfeeding positively impact the mother’s own emotions and feelings and elicit positive emotional responses in others? | 218 (42.50%) | 234 (45.61%) | 61 (11.89%) |
| 13. Most mothers can produce enough milk to meet their baby’s needs. | 201 (39.18%) | 251 (48.93%) | 61 (11.89%) |
| 14. Severe pain or emotional distress in the mother can inhibit the oxytocin reflex, causing milk secretion to suddenly stop and affecting the success of exclusive breastfeeding. | 209 (40.74%) | 244 (47.56%) | 60 (11.70%) |
| 15. If a mother stops lactating due to physical discomfort or emotional distress, milk production can resume with support and assistance, improved mood, and continued breastfeeding. | 207 (40.35%) | 257 (50.10%) | 49 (9.55%) |

Supplementary table 2. Attitude dimension of the participants.

| Attitude | **N (%)** | | | | |
| --- | --- | --- | --- | --- | --- |
|  | **Strongly believe** | **Believe** | **Neutral** | **Disbelieve** | **Strongly disbelieve** |
| 1. Do you believe that breastfeeding is the best feeding method for infants? | 194 (37.82%) | 164 (31.97%) | 76 (14.81%) | 36 (7.02%) | 43 (8.38%) |
| 2. Are you willing to overcome the additional challenges of maternal-infant separation to breastfeed? | 196 (38.21%) | 162 (31.58%) | 77 (15.01%) | 44 (8.58%) | 34 (6.63%) |
| 3. Do you agree that more support and resources from medical institutions can help mothers experiencing maternal-infant separation to continue breastfeeding? | 175 (34.11%) | 185 (36.06%) | 84 (16.37%) | 34 (6.63%) | 35 (6.82%) |
| 4. Do you agree that mothers experiencing maternal-infant separation need more understanding and support from society and family to continue breastfeeding? | 177 (34.50%) | 183 (35.67%) | 77 (15.01%) | 41 (7.99%) | 35 (6.82%) |
| 5. Has maternal-infant separation negatively impacted your ability to breastfeed? (open-ended questions) | 181 (35.28%) | 173 (33.72%) | 100 (19.49%) | 32 (6.24%) | 27 (5.26%) |
| 6. Do you believe that positive support from the daily environment, such as work environment (availability and length of breastfeeding leave), family members (husband, parents, in-laws), surrounding people (general public or friends), and public places will promote your breastfeeding decision? | 196 (38.21%) | 157 (30.60%) | 96 (18.71%) | 39 (7.60%) | 25 (4.87%) |
|  | Work environment | Family members | Surrounding people | Public places |  |
| 7. Which environment do you believe most affects your breastfeeding decision? (open-ended questions) | 207 (40.35%) | 197 (38.40%) | 51 (9.94%) | 58 (11.31%) | 0 |
| 8. Do you agree that postpartum visits and psychological adjustment can effectively improve anxiety, depression, or poor psychological state in mothers, helping them to better navigate the breastfeeding period? | 176 (34.31%) | 181 (35.28) | 79 (15.40%) | 36 (7.02%) | 41 (7.99%) |
| 9. Do you agree that the level of support from medical staff influences your breastfeeding decision? | 178 (34.70%) | 173 (33.72%) | 94 (18.32%) | 38 (7.41%) | 30 (5.85%) |
| 10. Would you be more willing and find it easier to breastfeed if there were professional guidance and intervention? | 170 (33.14%) | 196 (38.21%) | 80 (15.59%) | 30 (5.85%) | 37 (7.21%) |

Supplementary table 3. Practice dimension of the participants.

| Practice | **N (%)** | | | | | | |  |
| --- | --- | --- | --- | --- | --- | --- | --- | --- |
|  | **Strongly agree** | **Agree** | **Somewhat agree** | | | **Disagree** | **Strongly disagree** | |
| 1. During pregnancy (before delivery), did you actively seek breastfeeding information to learn about breastfeeding knowledge and skills? | 188 (36.65%) | 149 (29.04%) | 112 (21.83%) | | | 24 (4.68%) | 40 (7.80%) | |
|  | Breastfeeding | Infant formula | | | Mixed feeding |  |  | |
| 2. During pregnancy (before delivery), did you discuss and confirm the feeding method with your family? | 226 (44.05%) | 216 (42.11%) | | 71 (13.84%) | | / | / | |
|  | Breastfeeding | Infant formula | | Mixed feeding | |  |  | |
| 3. After the end of maternal-infant separation, what feeding method did you choose or hope to choose for your baby? | 210 (40.94%) | 218 (42.50%) | | 85 (16.57%) | | / | / | |
|  | Strongly agree | Agree | | Somewhat agree | | Disagree | Strongly disagree | |
| 4. During maternal-infant separation, did you receive regular professional physical massage (using lactation devices or professional lactation massage) to promote lactation and successfully achieve breastfeeding later? | 174 (33.92%) | 172 (33.53%) | | 105 (20.47%) | | 29 (5.65%) | 33 (6.43%) | |
| 5. During maternal-infant separation, did you use a breast pump or other methods to express milk regularly to empty your breasts? | 168 (32.75%) | 172 (33.53%) | | 105 (20.47%) | | 35 (6.82%) | 33 (6.43%) | |
| 6. During maternal-infant separation, did you regularly communicate with doctors or nurses to ensure smooth breastfeeding? | 175 (34.11%) | 169 (32.94%) | | 94 (18.32%) | | 39 (7.60%) | 36 (7.02%) | |
| 7. During separation from your baby, did you provide breast milk according to the regular feeding schedule? | 168 (32.75%) | 168 (32.75%) | | 105 (20.47%) | | 35 (6.82%) | 37 (7.21%) | |
| 8. If conditions allowed, did you (or will you) ensure your baby breastfeeds immediately after birth or after ending maternal-infant separation? | 177 (34.50%) | 169 (32.94%) | | 94 (18.32%) | | 40 (7.80%) | 33 (6.43%) | |
|  | My physical condition does not allow breastfeeding | I do not want breastfeed | | No response | |  |  | |
| 9. Reasons for not allowing your baby to breastfeed immediately: | 398 (77.58%) | 40 (7.80%) | | 75 (14.62%) | |  |  | |
|  |  |  | |  | |  |  | |
| 10. Have you paid more attention to your diet to ensure the quality of your breast milk? | 220 (42.88%) | 175 (34.11%) | | 51 (9.94%) | | 67 (13.06%) |  | |
| 11. After being reunited with your baby, did you (or will you) continue to breastfeed? | 186 (36.26%) | 208 (40.55%) | | 54 (10.53%) | | 65 (12.67%) |  | |
| 12. In terms of breastfeeding, did you (or will you) actively seek help from relevant professionals to increase the success rate of breastfeeding? | 193 (37.62%) | 192 (37.43%) | | 55 (10.72%) | | 73 (14.23%) |  | |
| 13. After delivery, did you (or will you) seek psychological adjustment or counseling to maintain a positive mindset for successful breastfeeding? | 219 (42.69%) | 168 (32.75%) | | 46 (8.97%) | | 80 (15.59%) |  | |

**Supplementary Table 4. Linear regression analysis for knowledge dimension**

|  | **Univariate linear regression** | | **Multivariate linear regression** | |
| --- | --- | --- | --- | --- |
|  | **Beta (95%CI)** | **P** | **Beta (95%CI)** | **P** |
| **Age** |  |  |  |  |
| <25 years old | -0.940(-3.007-1.128) | 0.372 |  |  |
| 25-30 years old | 0.886(-0.833-2.605) | 0.312 |  |  |
| 30-35 years old | -1.483(-3.373-0.408) | 0.124 |  |  |
| >35 years old | ref |  |  |  |
| **Education level** |  |  |  |  |
| High school or below | 1.501(-1.077-4.078) | 0.253 |  |  |
| Associate degree | 0.995(-0.846-2.837) | 0.289 |  |  |
| Bachelor’s degree | 0.464(-1.216-2.144) | 0.588 |  |  |
| Master’s degree or above | ref |  |  |  |
| **Your current employment status** |  |  |  |  |
| Housewife | 0.282(-3.170-3.734) | 0.873 |  |  |
| Employed | 0.057(-3.004-3.118) | 0.971 |  |  |
| Self-employed | 0.931(-2.289-4.151) | 0.570 |  |  |
| Other | ref |  |  |  |
| **Household’s disposable income (annual)** |  |  |  |  |
| 80000 yuan or above | -0.574(-2.113-0.966) | 0.465 |  |  |
| 40000-80000 yuan | 0.171(-1.480-1.822) | 0.839 |  |  |
| 16000-40000 | ref |  |  |  |
| **How many children do you have?** |  |  |  |  |
| 1 | 0.043(-1.197-1.283) | 0.946 |  |  |
| 2 or more | ref |  |  |  |
| **Your mode of delivery** |  |  |  |  |
| Vaginal delivery | -1.482(-2.729- -0.236) | 0.020 | -1.311(-2.553- -0.068) | 0.039 |
| Cesarean section | ref |  | ref |  |
| **Your current feeding method** |  |  |  |  |
| Exclusive breastfeeding | -13.344(-16.798- -9.890) | <0.001 |  |  |
| Mixed feeding | -11.383(-14.822- -7.944) | <0.001 |  |  |
| Formula feeding | ref |  |  |  |
| **Current postpartum period** |  |  |  |  |
| ≤3 days | ref |  |  |  |
| 3-7 days | 0.144(-1.759-2.046) | 0.882 |  |  |
| 7-42 days | -0.041(-2.105-2.022) | 0.969 |  |  |
| 42 days- 6 months | 1.469(-0.677-3.616) | 0.179 |  |  |
| Greater than 6 months | -0.004(-2.265-2.256) | 0.997 |  |  |
| **What stage are you currently in?** |  |  |  |  |
| Already experienced maternal-infant separation; currently, the separation has ended. | -2.055(-3.362- -0.749) | 0.002 | -1.926(-3.234- -0.618) | 0.004 |
| Currently experiencing maternal-infant separation; the separation has not yet ended. | ref |  | ref |  |

_Before conducting the multivariable regression analyses, multicollinearity diagnostics were performed for all independent variables. The results showed that the variance inflation factor (VIF) for feeding method in the attitude model exceeded 10, indicating substantial multicollinearity._

**Supplementary Table 5. Linear regression analysis for attitude dimension**

|  | **Univariate linear regression** | | **Multivariate linear regression** | |
| --- | --- | --- | --- | --- |
|  | **Beta (95%CI)** | **P** | **Beta (95%CI)** | **P** |
| K**nowledge dimension** | -0.946(-0.988- -0.904) | <0.001 | -0.941(-0.984- -0.898) | <0.001 |
| **Age** |  |  |  |  |
| <25 years old | 0.385(-1.824-2.594) | 0.732 |  |  |
| 25-30 years old | -0.941(-2.778-0.896) | 0.315 |  |  |
| 30-35 years old | 1.121(-0.899-3.140) | 0.276 |  |  |
| >35 years old | ref |  |  |  |
| **Education level** |  |  |  |  |
| High school or below | -1.132(-3.877-1.612) | 0.418 |  |  |
| Associate degree | -0.740(-2.701-1.221) | 0.459 |  |  |
| Bachelor’s degree | -0.754(-2.543-1.035) | 0.408 |  |  |
| Master’s degree or above | ref |  |  |  |
| **Your current employment status** |  |  |  |  |
| Housewife | -0.896(-4.563-2.770) | 0.631 |  |  |
| Employed | -0.263(-3.514-2.988) | 0.874 |  |  |
| Self-employed | -1.598(-5.018-1.822) | 0.359 |  |  |
| Other | ref |  |  |  |
| **Household’s disposable income (annual)** |  |  |  |  |
| 80000 yuan or above | 1.072(-0.564-2.707) | 0.199 |  |  |
| 40000-80000 yuan | 0.044(-1.711-1.798) | 0.961 |  |  |
| 16000-40000 | ref |  |  |  |
| **How many children do you have?** |  |  |  |  |
| 1 | 0.250(-1.068-1.569) | 0.709 |  |  |
| 2 or more | ref |  |  |  |
| **Your mode of delivery** |  |  |  |  |
| Vaginal delivery | 1.113(-0.217- 2.442) | 0.101 |  |  |
| Cesarean section | ref |  |  |  |
| **Your current feeding method** |  |  |  |  |
| Exclusive breastfeeding | 13.017(9.330- 16.704) | <0.001 |  |  |
| Mixed feeding | 10.338(6.668- 14.009) | <0.001 |  |  |
| Formula feeding | ref |  |  |  |
| **Current postpartum period** |  |  |  |  |
| ≤3 days | ref |  |  |  |
| 3-7 days | -0.532(-2.556-1.492) | 0.606 |  |  |
| 7-42 days | 0.075(-2.121-2.271) | 0.947 |  |  |
| 42 days- 6 months | -1.049(-3.333-1.235) | 0.367 |  |  |
| Greater than 6 months | 0.765(-1.640-3.170) | 0.532 |  |  |
| **What stage are you currently in?** |  |  |  |  |
| Already experienced maternal-infant separation; currently, the separation has ended. | 2.492(1.106- 3.878) | <0.001 | 0.558(-0.089- 1.205) | 0.091 |
| Currently experiencing maternal-infant separation; the separation has not yet ended. | ref |  | ref |  |

_Before conducting the multivariable regression analyses, multicollinearity diagnostics were performed for all independent variables. The results showed that the variance inflation factor (VIF) for feeding method in the attitude model exceeded 10, indicating substantial multicollinearity._

**Supplementary Table 6. Linear regression analysis for practice dimension**

|  | **Univariate linear regression** | | **Multivariate linear regression** | |
| --- | --- | --- | --- | --- |
|  | **Beta (95%CI)** | **P** | **Beta (95%CI)** | **P** |
| K**nowledge dimension** | -1.199(-1.256- -1.141) | <0.001 | -0.662(-0.777- -0.547) | <0.001 |
| A**ttitude dimension** | 1.119(1.064-1.174) | <0.001 | 0.560(0.451-0.668) | <0.001 |
| **Age** |  |  |  |  |
| <25 years old | 1.988(-0.835-4.810) | 0.167 |  |  |
| 25-30 years old | -1.095(-3.442-1.252) | 0.360 |  |  |
| 30-35 years old | 2.478(-0.103-5.058) | 0.060 |  |  |
| >35 years old | ref |  |  |  |
| **Education level** |  |  |  |  |
| High school or below | -1.431(-4.957-2.096) | 0.426 |  |  |
| Associate degree | -2.223(-4.743-0.297) | 0.084 |  |  |
| Bachelor’s degree | -1.672(-3.971-0.626) | 0.154 |  |  |
| Master’s degree or above | ref |  |  |  |
| **Your current employment status** |  |  |  |  |
| Housewife | -0.098(-4.829-4.633) | 0.967 |  |  |
| Employed | 0.433(-3.762-4.628) | 0.839 |  |  |
| Self-employed | -0.502(-4.915-3.911) | 0.823 |  |  |
| Other | ref |  |  |  |
| **Household’s disposable income (annual)** |  |  |  |  |
| 80000 yuan or above | 1.410(-0.691-3.511) | 0.188 |  |  |
| 40000-80000 yuan | -0.673(-2.926-1.580) | 0.557 |  |  |
| 16000-40000 | ref |  |  |  |
| **How many children do you have?** |  |  |  |  |
| 1 | -0.164(-1.862-1.534) | 0.850 |  |  |
| 2 or more | ref |  |  |  |
| **Your mode of delivery** |  |  |  |  |
| Vaginal delivery | 2.205(0.500- 3.910) | 0.011 | 0.570(-0.195-1.334) | 0.144 |
| Cesarean section | ref |  |  |  |
| **Your current feeding method** |  |  |  |  |
| Exclusive breastfeeding | 14.510(9.669- 19.350) | <0.001 |  |  |
| Mixed feeding | 12.569(7.750- 17.388) | <0.001 |  |  |
| Formula feeding | ref |  |  |  |
| **Current postpartum period** |  |  |  |  |
| ≤3 days | ref |  |  |  |
| 3-7 days | -0.720(-3.320-1.881) | 0.587 |  |  |
| 7-42 days | 0.003(-2.819-2.825) | 0.998 |  |  |
| 42 days- 6 months | -2.731(-5.666-0.204) | 0.068 |  |  |
| Greater than 6 months | -1.074(-4.165-2.016) | 0.495 |  |  |
| **What stage are you currently in?** |  |  |  |  |
| Already experienced maternal-infant separation; currently, the separation has ended. | 3.157(1.372- 4.943) | <0.001 | 0.345(-0.464- 1.155) | 0.403 |
| Currently experiencing maternal-infant separation; the separation has not yet ended. | ref |  | Ref |  |

_Before conducting the multivariable regression analyses, multicollinearity diagnostics were performed for all independent variables. The results showed that the variance inflation factor (VIF) for feeding method in the attitude model exceeded 10, indicating substantial multicollinearity._
