# Supplementary material for: Knowledge, attitude, and practice of breastfeeding among mothers experiencing maternal-infant separation: a cross-sectional study
Source: Front Public Health. 2026 Jun 16;14:1776575. doi: 10.3389/fpubh.2026.1776575 (PMC13315230; doi:10.3389/fpubh.2026.1776575)
Supplement: Supplementary file 2 [file Supplementary_File_1.doc]

Dear Mother,

Hello!

We are researchers from XX and we warmly invite you to participate in our research study. This study aims to collect information to enhance our understanding of breastfeeding among mothers who have experienced mother-infant separation. The data gathered will provide a foundation for developing scientific intervention strategies.

To participate in our research, you only need to complete this questionnaire based on your actual situation. Please be assured that the contents of the questionnaire will be kept confidential, and your information will not be disclosed.

We sincerely appreciate you taking the time out of your busy schedule to support our scientific research!

□ I am aware and agree that the collected data will be used for scientific research.

**Definition of ：Maternal Separation: Maternal separation (MS) refers to the separation between a young individual and their mother occurring from birth to weaning. Within the first six months after your child was born, did you experience a separation from your child for more than 24 hours?**

A: Yes

B: No

**Part 1 Basic Information**

1. Your Age：

a. ＜25 years old

b. 25 – 30 years old

c. 30 – 35 years old

d. ＞35 years old

2. Your Education level：

a. High school or below

b. Associate degree

c. Bachelor’s degree

d. Master’s degree

3. What is your current employment status：

a. Student

b. Housewife

c. Employed

d. Self-employed

e. Other (please specify):_______

4. What is your household’s disposable income (annual)?

a. 80000 yuan or above

b. 40000-80000 yuan

c. 16000-40000 yuan

d. Below 16000 yuan

5. How many children do you have?

a. 1

b. 2

c. ≥3

6. What is your mode of delivery?

a. Vaginal delivery

b. Cesarean section

7. What is your current feeding method?

a. Exclusive breastfeeding

b. Mixed feeding

c. Formula feeding

8. Your current postpartum period：

a. ≤3 days

b. 3~7 days

c. 7-42 days

d. 42 days-6 months

e．Greater than 6 months

11. What stage are you currently in?

a. Already experienced maternal separation; currently, the separation has ended.

b. Currently experiencing maternal separation; the separation has not yet ended.

**Part 2 Knowledge about Breastfeding**

Please indicate your level of understanding of the following statements:

1. **Exclusive breastfeeding is the best food for newborns.**

a. Very clear

b. Heard of it

c. Never heard of it

**2. Breast milk contains sufficient energy and nutrients, which are more easily digested and absorbed.**

a. Very clear

b. Heard of it

c. Never heard of it

**3. The optimal feeding method for infants is exclusive breastfeeding from birth to 6 months, continuing breastfeeding up to 2 years or beyond.**

a. Very clear

b. Heard of it

c. Never heard of it

**4. Breastfeeding can enhance the emotional bond between the child and the mother, providing warmth and love to the child.**

a. Very clear

b. Heard of it

c. Never heard of it

**5. Can breastfeeding improve a child’s immunity?**

a. Very clear

b. Heard of it

c. Never heard of it

**6. Breastfed children are less likely to develop diabetes, heart disease, eczema, asthma, rheumatoid arthritis, and other allergic diseases, and breastfeeding can prevent obesity.**

a. Very clear

b. Heard of it

c. Never heard of it

**7.Can a mother continue breastfeeding her baby if she has the flu or a severe cold?**

a. Very clear

b. Heard of it

c. Never heard of it

**8.Breastfeeding can reduce the risk of breast and ovarian cancer in women.**

a. Very clear

b. Heard of it

c. Never heard of it

**9.Breastfeeding can reduce postpartum bleeding and anemia, promoting faster recovery after childbirth.**

a. Very clear

b. Heard of it

c. Never heard of it

**10. Breastfeeding mothers are less likely to be obese; breastfeeding helps mothers return to their normal body shape.**

a. Very clear

b. Heard of it

c. Never heard of it

**11.Exclusive breastfeeding has a contraceptive effect, inhibiting ovulation and delaying the return of fertility.**

a. Very clear

b. Heard of it

c. Never heard of it

**12.Can exclusive breastfeeding positively impact the mother’s own emotions and feelings and elicit positive emotional responses in others?**

a. Very clear

b. Heard of it

c. Never heard of it

**13. Most mothers can produce enough milk to meet their baby’s needs.**

a. Very clear

b. Heard of it

c. Never heard of it

**14. Severe pain or emotional distress in the mother can inhibit the oxytocin reflex, causing milk secretion to suddenly stop and affecting the success of exclusive breastfeeding.**

a. Very clear

b. Heard of it

c. Never heard of it

**15 .If a mother stops lactating due to physical discomfort or emotional distress, milk production can resume with support and assistance, improved mood, and continued breastfeeding.**

a. Very clear

b. Heard of it

c. Never heard of it

**Part 3 Attitude towards Breastfeeding**

**1. Do you believe that breastfeeding is the best feeding method for infants?**

a. Strongly believe

b. Believe

c. Neutral

d. Disbelieve

e. Strongly disbelieve

**2. Are you willing to overcome the additional challenges of maternal separation to breastfeed?**

a. Very willing

b. Willing

c. Neutral

d. Unwilling

e. Strongly unwilling

**3.Do you agree that more support and resources from medical institutions can help mothers experiencing maternal separation to continue breastfeeding?**

a. Strongly agree

b. Agree

c. Neutral

d. Disagree

e. Strongly disagree

**4. Do you agree that mothers experiencing maternal separation need more understanding and support from society and family to continue breastfeeding?**

a. Strongly agree

b. Agree

c. Neutral

d. Disagree

e. Strongly disagree

**5. Has maternal separation negatively impacted your ability to breastfeed?**

a. Strongly agree

b. Agree

c. Neutral

d. Disagree

e. Strongly disagree

**6. Do you believe that positive support from the daily environment, such as work environment (availability and length of breastfeeding leave), family members (husband, parents, in-laws), surrounding people (general public or friends), and public places will promote your breastfeeding decision?**

a. Strongly agree

b. Agree

c. Neutral

d. Disagree

e. Strongly disagree

**7.Which environment do you believe most affects your breastfeeding decision?**

a．Work environment

b．Family members

c．Surrounding people

d．Public places

**8. Do you agree that postpartum visits and psychological adjustment can effectively improve anxiety, depression, or poor psychological state in mothers, helping them to better navigate the breastfeeding period?**

a. Strongly agree

b. Agree

c. Neutral

d. Disagree

e. Strongly disagree

**9. Do you agree that the level of support from medical staff influences your breastfeeding decision?**

a. Strongly agree

b. Agree

c. Neutral

d. Disagree

e. Strongly disagree

**10. Would you be more willing and find it easier to breastfeed if there were professional guidance and intervention?**

a. Strongly agree

b. Agree

c. Neutral

d. Disagree

e. Strongly disagree

**Part 4 Practice of Breastfeeding Decisions**

**1. During pregnancy (before delivery), did you actively seek breastfeeding information to learn about breastfeeding knowledge and skills?**

A.Strongly agree

B.Agree

C.Somewhat agree

D.Disagree

E.Strongly disagree

**2. During pregnancy (before delivery), did you discuss and confirm the feeding method with your family?**

A. Breastfeeding

B. Infant formula

C. Mixed feeding

**3. After the end of maternal separation, what feeding method did you choose or hope to choose for your baby?**

A. Breastfeeding

B. Infant formula

C. Mixed feeding

**4. During maternal separation, did you receive regular professional physical massage (using lactation devices or professional lactation massage) to promote lactation and successfully achieve breastfeeding later?**

A.Strongly agree

B.Agree

C.Somewhat agree

D.Disagree

E.Strongly disagree

**5. During maternal separation, did you use a breast pump or other methods to express milk regularly to empty your breasts?**

A.Strongly agree

B.Agree

C.Somewhat agree

D.Disagree

E.Strongly disagree

**6. During maternal separation, did you regularly communicate with doctors or nurses to ensure smooth breastfeeding?**

A.Strongly agree

B.Agree

C.Somewhat agree

D.Disagree

E.Strongly disagree

**7. During separation from your baby, did you provide breast milk according to the regular feeding schedule?**

A.Strongly agree

B.Agree

C.Somewhat agree

D.Disagree

E.Strongly disagree

**8.If conditions allowed, did you (or will you) ensure your baby breastfeeds immediately after birth or after ending maternal separation?**

A.Strongly agree

B.Agree

C.Somewhat agree

D.Disagree

E.Strongly disagree

**9. Reasons for not allowing your baby to breastfeed immediately: (selective response)**

A. My physical condition does not allow breastfeeding

B. I do not want to breastfeed

10. **Have you paid more attention to your diet to ensure the quality of your breast milk?**

A.Strongly agree

B.Agree

C.Somewhat agree

D.Disagree

**11.** **After being reunited with your baby, did you (or will you) continue to breastfeed?**

A.Strongly agree

B.Agree

C.Somewhat agree

D.Disagree

**12. In terms of breastfeeding, did you (or will you) actively seek help from relevant professionals to increase the success rate of breastfeeding?**

A.Strongly agree

B.Agree

C.Somewhat agree

D.Disagree

**13.** **After delivery, did you (or will you) seek psychological adjustment or counseling to maintain a positive mindset for successful breastfeeding?**

A.Strongly agree

B.Agree

C.Somewhat agree

D.Disagree
